# Aspartame and cardiovascular disease: Unraveling potential molecular mechanisms through integrative network toxicology, molecular docking, and dynamics simulation

**DOI:** 10.1097/MD.0000000000046012

**Published:** 2025-11-21

**Authors:** Taoyu Yang, Jiangting Luo, Lili Zhang, Haowei Li, Jingjing Wang

**Affiliations:** a First Clinical Medical College, General Hospital of Ningxia Medical University, Yinchuan, China; b Department of Cardiology, General Hospital of Ningxia Medical University, Yinchuan, China.

**Keywords:** aspartame toxicity, cardiovascular disease, molecular docking, network toxicology

## Abstract

Aspartame, a widely used artificial sweetener, has raised growing concerns regarding its potential cardiovascular toxicity. While regulatory agencies deem it safe within established limits, emerging evidence suggests possible adverse effects on vascular and inflammatory systems. This study aimed to investigate the potential molecular mechanisms by which aspartame may contribute to cardiovascular disease, utilizing a network toxicology approach combined with molecular docking and dynamics simulation. Potential aspartame targets were predicted using ProTox 3.0 and ADMETlab 2.0 platforms, alongside ChEMBL, STITCH, and Swiss Target Prediction databases. Cardiovascular-related targets were identified via GeneCards, Online Mendelian Inheritance in Man, and the Therapeutic Target Database. Overlapping genes were analyzed through Gene Ontology and Kyoto Encyclopedia of Genes and Genomes pathway enrichment. A protein–protein interaction network was constructed and analyzed for hub gene identification. Molecular docking and 100 ns molecular dynamics simulations were performed to validate binding stability between aspartame and key targets. Fifty-three overlapping genes were identified between aspartame and cardiovascular disease-related targets. Three hub proteins – interleukin-1β, caspase-3, and SRC – were revealed as potential regulators of aspartame-induced cardiovascular effects. Aspartame demonstrated stable binding to these proteins, particularly CASP3. Functional enrichment highlighted the AGE-RAGE, NF-κB, and PI3K-Akt signaling pathways as key mediators. Our findings suggest that aspartame may influence cardiovascular health through coordinated modulation of inflammatory and apoptotic pathways. These results provide a molecular framework for further experimental validation and risk stratification in sensitive populations.

## 1. Introduction

Aspartame is an artificial dipeptide sweetener composed primarily of phenylalanine and aspartic acid.^[1]^ It is approximately 200 times sweeter than sucrose and contains negligible calories, thus widely used in food and beverage products as a low-calorie sugar substitute.^[2]^ Regulatory agencies such as the United States Food and Drug Administration (FDA) and the European Food Safety Authority have established acceptable daily intake (ADI) limits for aspartame, set at 50 mg/kg body weight in the United States and 40 mg/kg in Europe.^[3]^ Although aspartame may help reduce added sugar intake, long-term consumption can potentially exceed the FDA-defined ADI, raising concerns about its long-term safety, particularly regarding its potential association with metabolic disorders including obesity, diabetes, and cardiovascular diseases (CVD).^[4–7]^ Some studies suggest that aspartame’s metabolites such as phenylalanine may impair endothelial function by interfering with nitric oxide (NO) signaling, while others propose that methanol may contribute to oxidative stress, potentially damaging vascular function and increasing CVD risk.^[8]^ Moreover, potential neurotoxic effects of aspartame, especially those involving neuroinflammation and oxidative stress, may further amplify cardiovascular risk, given the tight coupling between the brain and vascular systems.^[9]^ Recent studies have also applied network toxicology and molecular docking to explore aspartame’s potential links to other diseases. For instance, Zhang et al identified key targets and pathways linking aspartame to ischemic stroke,^[9]^ while Yang et al and Chen et al investigated its potential role in female infertility^[10]^ and carcinogenicity,^[11]^ respectively. These studies support the utility of computational approaches in hypothesizing aspartame’s systemic effects.

Preclinical and clinical evidence indicates that aspartame may be a potential CVD risk factor by influencing hypertension, vascular dysfunction, inflammation, weight gain, and gut microbiota alterations.^[12–14]^ The large-scale NutriNet-Santé prospective cohort study, involving 103,388 participants, reported a possible direct association between increased artificial sweetener intake (particularly aspartame, acesulfame-K, and sucralose) and elevated cardiovascular risk.^[7]^ Clinical studies targeting specific populations have also found correlations between aspartame intake and increased coronary plaque burden and inflammatory markers, suggesting potential mechanisms linking aspartame and cardiovascular risk.^[15]^

ProTox 3.0 is a web-based toxicity prediction platform capable of estimating 61 types of toxicity endpoints, including acute toxicity, organ toxicity, and pathways such as Toxicology in the 21st Century (Tox21).^[16]^ ADMETlab 2.0 (absorption, distribution, metabolism, excretion, and toxicity [ADMET]) is a highly integrated online tool widely used for predicting the pharmacokinetics and toxicological profiles of chemical compounds.^[17]^ In this study, we utilized these 2 complementary prediction platforms to investigate aspartame’s toxicity and absorption in the human body, focusing on target identification and toxicity prediction.^[18,19]^ By integrating data from multiple databases, we constructed a complex network connecting toxicological targets, disease-associated targets, and biological processes.^[20]^ We found that aspartame may promote the progression of CVD by modulating specific immune-inflammatory factors. These findings enhance our understanding of the molecular mechanisms by which aspartame may influence CVD and highlight the potential of network toxicology in assessing health risks associated with synthetic additives.

## 2. Materials and methods

### 2.1. Study design

**Figure 1.**
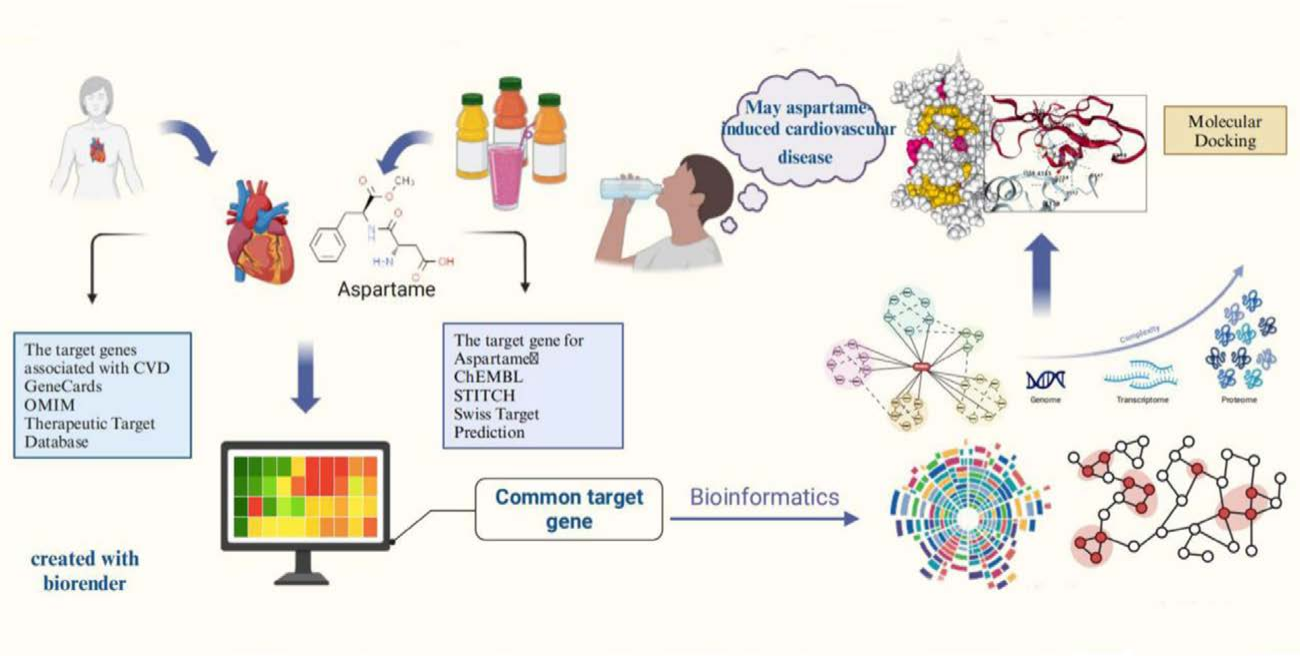
Bioinformatics analysis pipeline for investigating the interaction between aspartame and cardiovascular disease-related genes.

Toxicity prediction platforms were used to analyze the toxicity profile of aspartame and identify its potential targets. CVD-related genes were retrieved from the GeneCards database. Overlapping genes between the two sets were visualized using the ggvenn package in R. These overlapping targets were submitted to the Search Tool for the Retrieval of Interacting Genes/Proteins (STRING) database to construct a protein–protein interaction (PPI) network. Kyoto Encyclopedia of Genes and Genomes (KEGG) and Gene Ontology (GO) enrichment analyses were performed to elucidate the biological functions and pathways associated with the intersecting genes. The PPI network was visualized using Cytoscape, and centrality analysis was conducted using 4 different metrics. Core genes were further filtered using the CentiScaPe and Molecular Complex Detection (MCODE) plugins. Finally, molecular docking and molecular dynamics (MD) simulations were performed to assess the stability of interactions between aspartame and the identified cardiovascular targets. The workflow of this study is illustrated in Figure 1.

### 2.2. Pharmacokinetic properties and toxicity prediction of aspartame

The molecular structure and Simplified Molecular Input Line Entry System representation of aspartame (PubChem CID: 134601) were obtained from the PubChem database (https://pubchem.ncbi.nlm.nih.gov/). ADMET properties and toxicity profiles were assessed using the ADMETlab 2.0 platform (https://admetmesh.scbdd.com/) and the ProTox 3.0 server (https://tox.charite.de/protox3/). A probability threshold > 0.7 was considered as the criterion for positive toxicity prediction, with a focus on cardiovascular-related toxicity endpoints.

### 2.3. Target identification for aspartame

Based on aspartame’s molecular structure, potential protein targets were predicted using the Chemical Database of Bioactive Molecules with Drug-Like Properties (ChEMBL; https://www.ebi.ac.uk/chembl/), the Search Tool for Interacting Chemicals (STITCH; http://stitch.embl.de/), and Swiss Target Prediction (http://www.swisstargetprediction.ch/). In ChEMBL, bioactivity data with a pChEMBL value ≥ 6.0 (corresponding to IC50 or Ki ≤ 1 μM) were selected, limited to Homo sapiens. STITCH predictions based on structural similarity used a confidence score ≥ 0.4. Swiss Target Prediction applied 2D and 3D similarity algorithms with a probability threshold > 0.1. Predicted results from all 3 platforms were integrated using R (v4.4.1), and Venn diagrams were plotted using the ggvenn package.

### 2.4. Collection of CVD-associated targets

Cardiovascular-related genes were retrieved from GeneCards (https://www.genecards.org/), the Online Mendelian Inheritance in Man (OMIM; https://www.omim.org/), and the Therapeutic Target Database (TTD; http://db.idrblab.net/ttd/). In GeneCards, the keyword *cardiovascular disease* was used with a relevance score threshold ≥ 10. OMIM entries were screened using keywords such as *cardiovascular*, *heart disease*, *vascular disease*, and *cardiac*. TTD provided therapeutic targets including those approved or under clinical trials. All gene sets were consolidated and visualized using ggvenn.

### 2.5. Identification of core targets and construction of PPI network

The intersection of aspartame-related targets and CVD targets was computed using R to identify core targets. These were then submitted to the STRING database (https://cn.string-db.org) to construct a PPI network. Parameters were set to Homo sapiens and a minimum interaction score of 0.4 (medium confidence). The network was visualized and topologically analyzed using Cytoscape (v3.9.1, https://cytoscape.org/).

### 2.6. GO and pathway enrichment analysis

The clusterProfiler package (v4.6.0) in R was used to perform GO and KEGG enrichment analyses on core targets. A corrected *P*-value < .05 was considered statistically significant. The minimum and maximum gene set sizes were set to 5 and 500, respectively. GO analysis included biological process, cellular component, and molecular function. KEGG analysis was based on the latest pathway annotations.

### 2.7. Identification of hub genes

Topological features of the PPI network were analyzed using 4 centrality algorithms: betweenness centrality, closeness centrality, edge percolated component, and maximal clique centrality. The cytoHubba plugin in Cytoscape was used to calculate centrality scores, and the top 10 genes from each algorithm were selected. Their intersections were analyzed using the VennDiagram package in R (v4.4.1) to identify hub genes ranked within the top 10 by all 4 methods. Overlapping targets were further filtered using Venn plots.

### 2.8. Molecular docking between aspartame and core targets

Three-dimensional crystal structures of key target proteins were downloaded from the Research Collaboratory for Structural Bioinformatics Protein Data Bank (https://www.rcsb.org), selecting high-quality structures with resolutions < 2.5 Å. Water molecules, cofactors, and original ligands were removed using PyMOL, and polar hydrogens were added. Aspartame’s 3D structure (CID: 134601) was downloaded from PubChem and energy-minimized using MM2 force field in ChemBio3D Ultra or PyMOL. AutoDock Vina was used for docking,^[21,22]^ with a grid box size of 20 × 20 × 20 Å around the active site and exhaustiveness set to 8, generating 9 conformations. The best conformation was selected based on binding affinity and pose reasonableness.

### 2.9. Molecular dynamics simulations

GROMACS 2022.3 was used for MD simulations.^[23,24]^ AMBER14SB and general AMBER force field were used as force fields for proteins and ligands, respectively, with TIP3P as the solvent model. Built-in GROMACS tools were used to analyze root mean square deviation, root mean square fluctuation, radius of gyration (Rg), number of hydrogen bonds, and solvent-accessible surface area. Principal component analysis was conducted to assess conformational changes. Molecular mechanics Poisson–Boltzmann surface area binding free energy was calculated using g_mmpbsa, including van der Waals, electrostatic, polar, and nonpolar solvation energies, as well as residue-level energy contributions.

## 3. Results

### 3.1. ADMET properties and toxicity prediction of aspartame

**Figure 2.**
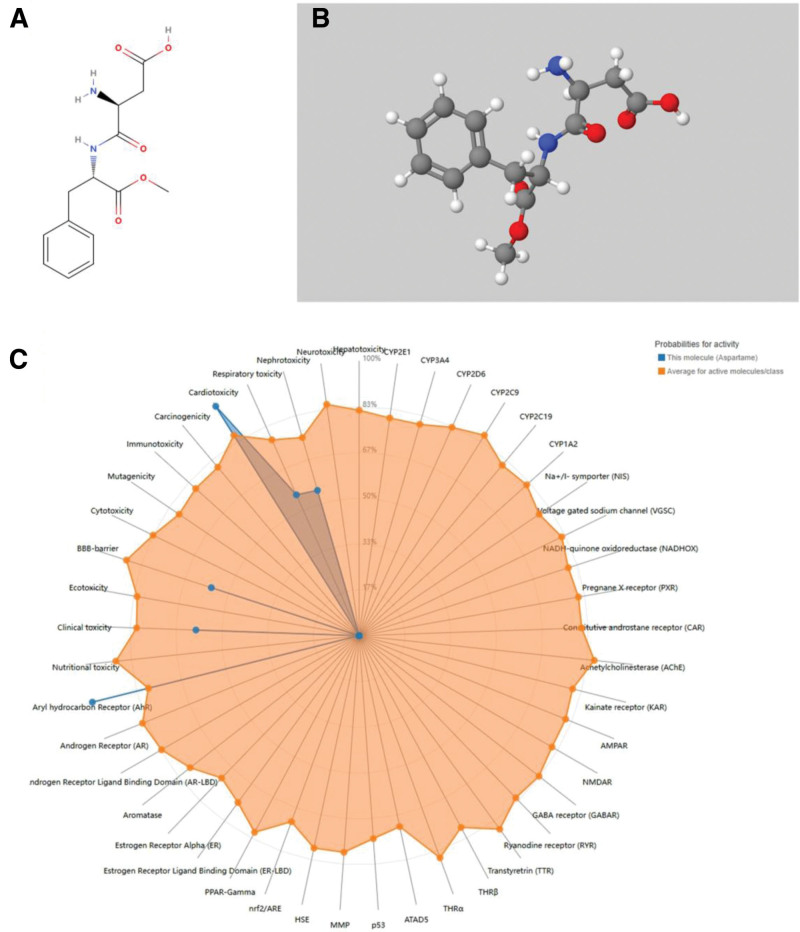
Molecular structure and predicted toxicity profile of aspartame. (A) Two-dimensional (2D) structure of aspartame. (B) Three-dimensional (3D) structure of aspartame. (C) Predicted ADMET properties and toxicity endpoints generated using ADMETlab 2.0 and ProTox 3.0. ADMET = absorption, distribution, metabolism, excretion, and toxicity.

Aspartame’s chemical structure (Fig. 2A, B) was evaluated for ADMET properties and toxicity using ADMETlab 2.0 and ProTox 3.0. ADMETlab 2.0 analysis revealed moderate blood–brain barrier penetration (probability: 0.68) and potential respiratory toxicity (0.643), while indicating low human ether-à-go-go-related gene (hERG)-related cardiotoxicity risk (0.022). In contrast, ProTox 3.0 predicted substantially higher general cardiotoxicity (0.98), suggesting cardiovascular effects through mechanisms beyond hERG channel blockade. Additional predictions included nephrotoxicity (0.55), respiratory toxicity (0.56), and complete aryl hydrocarbon receptor (AhR) activation (1.0; Fig. 2C). These preliminary findings prompted further database screening to identify cardiovascular-related targets for molecular docking and dynamics simulation.

### 3.2. Identification of aspartame-associated cardiovascular targets

**Figure 3.**
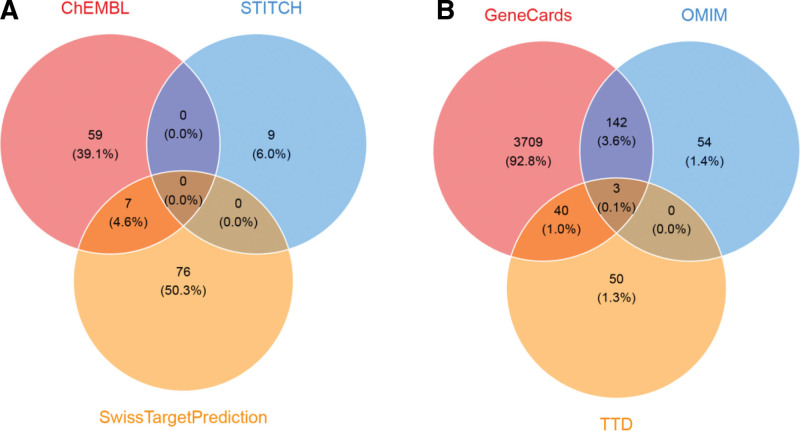
Identification of potential cardiovascular disease (CVD) targets associated with aspartame. (A) Venn diagram showing the unique and shared targets identified from ChEMBL, STITCH, and Swiss Target Prediction databases. (B) Venn diagram illustrating the unique and shared targets from GeneCards, OMIM, and TTD databases related to CVD. OMIM = Online Mendelian Inheritance in Man, STITCH = Search Tool for Interacting Chemicals, TTD = Therapeutic Target Database.

**Figure 4.**
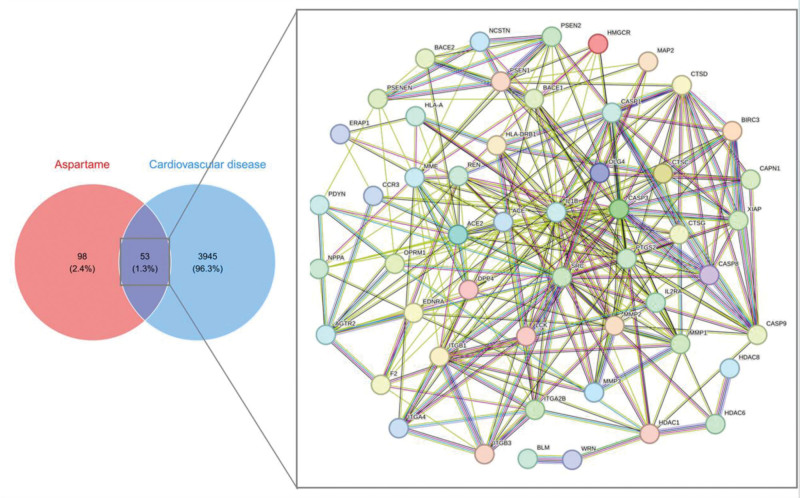
Aspartame–CVD target intersection and network. Venn diagram showing 53 shared targets between aspartame and CVD. PPI network of shared targets, highlighting potential collaborative roles in CVD pathology. CVD = cardiovascular disease, PPI = protein–protein interaction.

A total of 151 aspartame-related targets were identified from ChEMBL, STITCH, and Swiss Target Prediction (Fig. 3A). Simultaneously, 3998 cardiovascular-related targets were compiled from GeneCards, OMIM, and TTD (Fig. 3B). Intersection analysis (Fig. 4) revealed 53 overlapping targets, considered potential mediators of aspartame-induced cardiovascular toxicity.

### 3.3. PPI network construction

The 53 overlapping targets were input into STRING to construct the PPI network (Fig. 4), which consisted of 53 nodes and 276 edges, with an average node degree of 10.4 and a clustering coefficient of 0.52. The network diameter was 4. PPI enrichment yielded a *P*-value < 1.0 × 10^−16^, suggesting significant biological relevance among the interacting proteins.

### 3.4. Functional enrichment analysis

**Figure 5.**
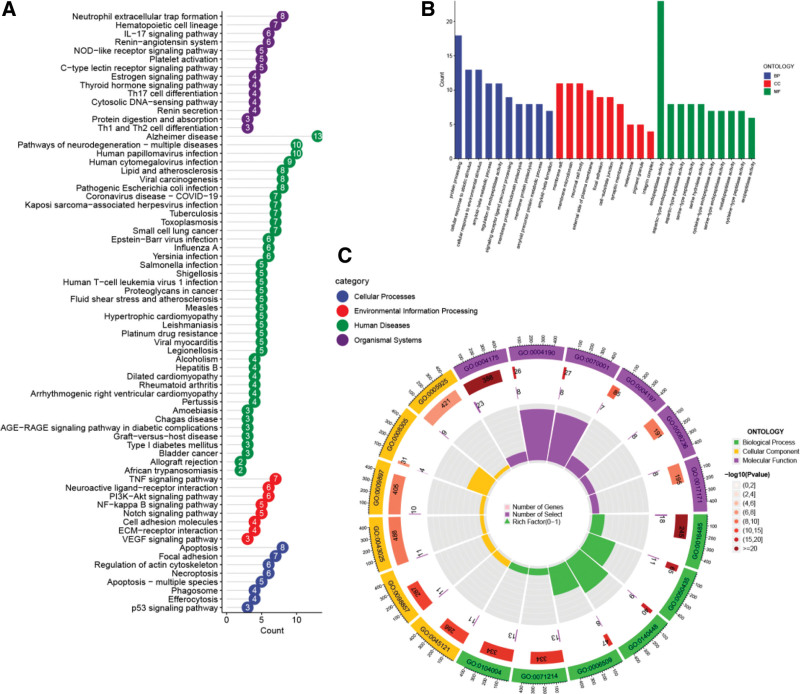
The top enriched KEGG pathways (A) and Gene Ontology terms (B–C) for the 53 shared targets. Bubble size indicates gene count, and color intensity reflects −log10(*P*-value). Key pathways include AGE-RAGE, NF-κB, PI3K-Akt, and apoptosis signaling. KEGG = Kyoto Encyclopedia of Genes and Genomes.

GO enrichment (Fig. 5B, C) indicated that core targets were involved in neutrophil extracellular trap formation, inflammation regulation, apoptosis, extracellular matrix remodeling, and oxidative stress. KEGG pathway analysis (Fig. 5A) identified 42 enriched pathways, including AGE-RAGE signaling in diabetic complications, NF-κB, PI3K-Akt, apoptosis, actin cytoskeleton regulation, platelet activation, estrogen, and thyroid hormone signaling. These pathways are closely associated with CVD mechanisms.

### 3.5. Identification of hub genes

**Figure 6.**
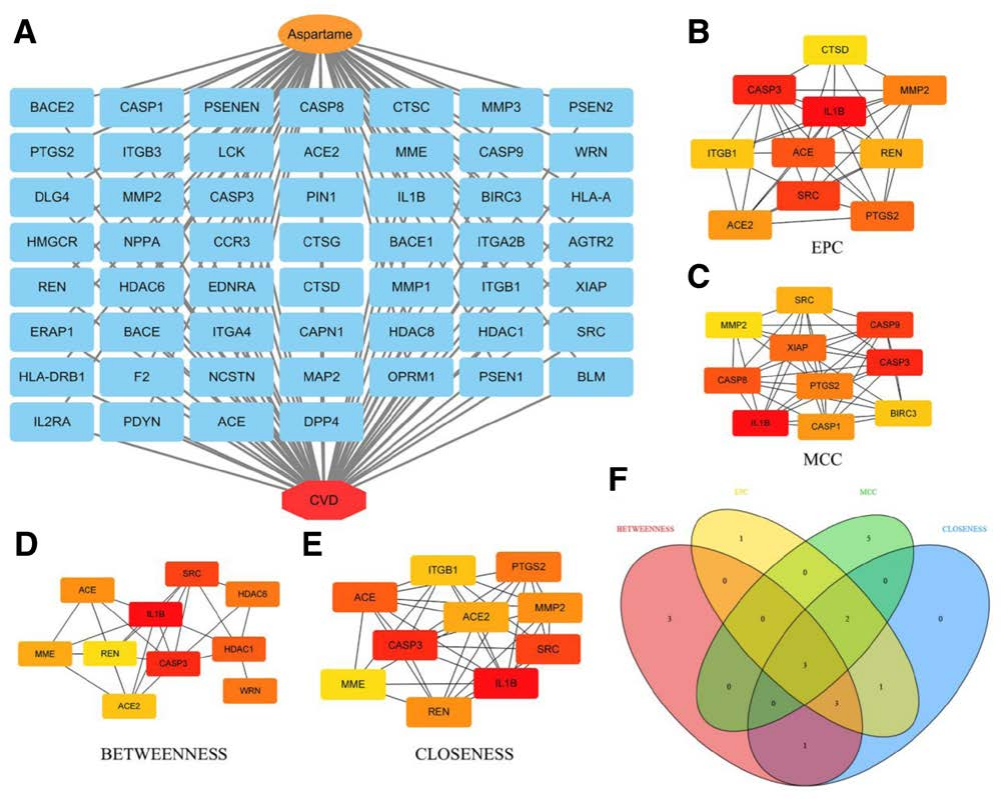
Identification of hub genes from the PPI network. (A–E) Top 10 genes ranked by 4 centrality metrics. (F) Venn diagram showing the 3 overlapping hub genes: IL1B, CASP3, and SRC. PPI = protein–protein interaction. Created in BioRender (https://BioRender.com/a31a061).

Four centrality algorithms identified the top 10 genes within the PPI network (Fig. 6A–E). Venn analysis (Fig. 6F) identified 3 overlapping hub genes – interleukin-1β (IL1B), CASP3 (caspase-3), and SRC (proto-oncogene tyrosine-protein kinase Src) – as key regulators of aspartame-related cardiovascular toxicity.

### 3.6. Molecular docking between aspartame and key targets

**Figure 7.**
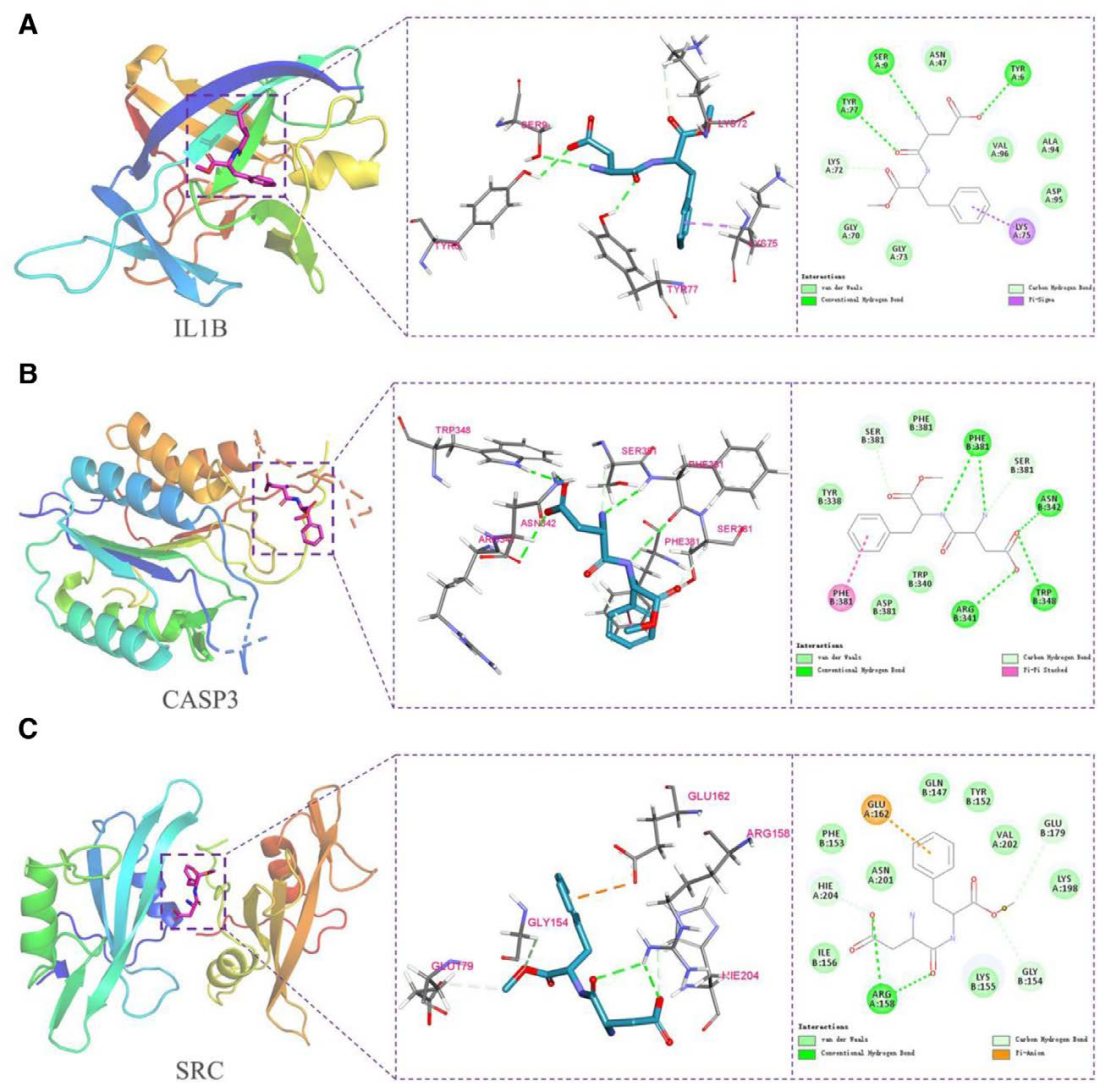
Molecular docking of aspartame (stick) with (A) IL1B, (B) CASP3, and (C) SRC. Dashed lines show hydrogen bonds and π–stacking interactions.

Molecular docking (Fig. 7) showed favorable binding affinities between aspartame and IL1B, CASP3, and SRC. All docking energies were below − 5.0 kcal/mol, indicating potential stability and functional relevance. Aspartame showed the strongest interaction with CASP3 (−6.3 kcal/mol; Table 1).

**Table 1:** Docking scores for aspartame with CASP3, SRC, and IL1B.

Target	PDB number	Hydrone	Affinity (kcal/mol)
CASP3	1re1	Aspartame	−6.3
SRC	1a07	Aspartame	−6.2
IL1B	4 × 37	Aspartame	−5.7

PDB = Protein Data Bank.

### 3.7. Molecular dynamics simulation

**Figure 8.**
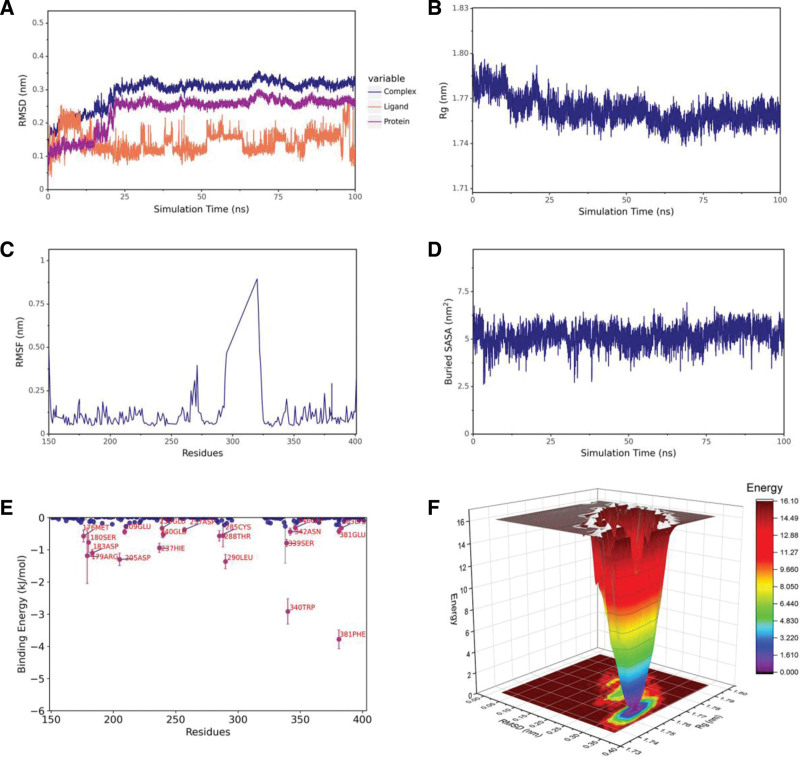
Hundred ns molecular dynamics simulation of the aspartame–CASP3 complex. (A) RMSD of protein (black), ligand (red), and complex (blue). (B) Radius of gyration (Rg). (C) RMSF per residue. (D) MM-PBSA binding energy contributions. (E) Residue free energy decomposition. (F) Mutation hotspot analysis (volcano plot). MM-PBSA = molecular mechanics Poisson–Boltzmann surface area, RMSD = root mean square deviation, RMSF = root mean square fluctuation.

A 100 ns MD simulation of the aspartame–CASP3 complex confirmed docking stability. Root mean square deviation stabilized around 25 ns (Fig. 8A), and Rg remained stable (Fig. 8B), indicating structural compactness. Root mean square fluctuation and solvent-accessible surface area (Fig. 8C–D) revealed flexible yet consistent binding. Residue contribution analysis showed PHE-381 and TRP-340 in CASP3 contributed significantly (Fig. 8E). Volcano mapping identifies a stabilizing pocket that constitutes the primary hotspot for mutations aimed at enhancing aspartame–CASP3 affinity (Fig. 8F). Molecular mechanics Poisson–Boltzmann surface area results (Table 2) indicated electrostatic interactions dominated IL1B binding, van der Waals interactions dominated CASP3 binding, and both forces contributed to SRC binding.

**Table 2:** MM-PBSA binding free energy analysis between aspartame and key targets.

Complex	ΔEvdw (kJ/mol)	ΔEele (kJ/mol)	ΔEMMPBSA (kJ/mol)
Aspartame–IL1B	−37.436 ± 9.191	−56.218 ± 3.704	43.104 ± 9.392
Aspartame–CASP3	−81.099 ± 3.275	−54.13 ± 1.054	16.302 ± 2.893
Aspartame–SRC	−62.052 ± 7.050	−57.302 ± 3.984	29.412 ± 5.865

MM-PBSA = molecular mechanics Poisson–Boltzmann surface area.

## 4. Discussion

This study employed a network toxicology strategy to systematically predict the potential molecular interactions between aspartame and the cardiovascular system, identifying IL1B, CASP3 (caspase-3), and SRC (proto-oncogene tyrosine-protein kinase Src) as key regulatory targets. It is essential to emphasize that these computational predictions serve as a scientific hypothesis-generation tool and theoretical analysis framework, rather than a challenge to the established safety of aspartame. Aspartame has been used globally for over 4 decades and has received extensive safety validation from multiple international regulatory agencies.^[25]^ However, its precise mechanisms of action, particularly at the molecular and systemic levels, remain incompletely understood. The application of network toxicology in this study aims to bridge this gap and offer supplementary molecular-level insights for conventional toxicological assessments.

Initial ADMET and toxicity predictions revealed notable cardiovascular concerns. A striking discrepancy emerged between ProTox 3.0’s substantially elevated cardiotoxicity risk and ADMETlab 2.0’s minimal hERG-related risk, suggesting aspartame’s cardiovascular effects may involve mechanisms beyond direct ion channel blockade. Particularly noteworthy was the complete AhR activation (1.0), as AhR signaling can promote cardiovascular toxicity through oxidative stress generation and NF-κB cascade activation – mechanisms consistent with inflammatory and apoptotic pathway disruptions observed in our enrichment analyses.^[26,27]^ The predictions also indicated moderate blood–brain barrier penetration capability alongside respiratory and renal toxicity risks, suggesting potential systemic effects. Collectively, these computational toxicology findings highlighted the necessity of investigating specific cardiovascular-related molecular targets.

IL1B, a central driver of inflammatory cascades, plays a pivotal role in cardiovascular inflammation.^[28]^ Molecular simulations confirmed the stable binding between aspartame and IL1B. Previous animal studies have reported that ADI levels of aspartame can elevate IL-1β expression in the gastrointestinal and nervous systems.^[29,30]^ While these findings mainly pertain to noncardiac systems, the crucial role of IL-1β in cardiovascular inflammation^[28,31]^ warrants further attention to its potential alterations within the cardiovascular system. The significant enrichment of the NF-κB signaling pathway and the prominent involvement of IL-1β in cardiovascular pathology suggest that the interaction between aspartame and IL1B requires deeper mechanistic investigation.

Caspase-3 (CASP3), a critical executioner of apoptosis, is implicated in cardiomyocyte death and myocardial injury. Molecular docking revealed that aspartame has the strongest binding affinity with CASP3. Experimental studies have observed increased CASP3 expression in mice following prolonged high-dose aspartame exposure,^[32]^ while chronic administration in rats at FDA-approved ADI (40 mg/kg) also upregulated Bcl-2 and CASP3, activating apoptotic pathways.^[33,34]^ The molecular modeling results suggest that aspartame may influence CASP3 enzymatic activity or conformational stability, potentially disrupting apoptosis balance. Given CASP3’s dual role in cardiovascular health – where moderate apoptosis clears damaged cells, but excessive activation may lead to substantial cardiomyocyte loss^[35]^ – it is critical to determine whether and how aspartame modulates CASP3 activity.

SRC, a non-receptor tyrosine kinase, regulates cell adhesion, migration, and vascular remodeling, and is essential for maintaining cardiovascular homeostasis.^[36]^ Molecular docking demonstrated stable interactions between aspartame and SRC. Disruption of SRC function may impair vascular remodeling,^[37]^ lead to endothelial dysfunction,^[38]^ promote atherosclerosis,^[39]^ and exacerbate reperfusion injury.^[40]^ Although there is currently no in vivo evidence showing that aspartame directly alters SRC expression, its predicted binding capacity and the pivotal role of SRC in vascular stability underscore the need to explore aspartame’s potential interference with SRC-mediated signaling.

IL1B, CASP3, and SRC interact within a complex signaling network that may generate multilayered synergistic effects. These targets serve as regulatory hubs within interconnected pathways.^[41]^ Our results suggest that aspartame may simultaneously engage upstream (IL1B) and downstream (CASP3) components of the inflammation-apoptosis axis, potentially amplifying their combined effects. IL-1β can activate MyD88-dependent signaling via its receptor, leading to NF-κB activation^[42,43]^ This in turn upregulates downstream inflammatory cytokines (e.g., TNF-α, IL-6) and induces Bcl-2 expression,^[44]^ ultimately influencing CASP3 activation. Additionally, IL1B may activate SRC,^[45]^ which then phosphorylates adhesion and tight junction proteins (vascular endothelial cadherin, β-catenin, occludin, and claudin), increasing vascular permeability.^[36]^ These mechanisms align with GO enrichment findings involving vascular development and cell migration, providing molecular insight into aspartame’s potential endothelial effects.

KEGG enrichment identified significant involvement of the AGE-RAGE and PI3K/Akt signaling pathways, both of which are central to cardiovascular pathology. Advanced glycation end products binding to their receptor for advanced glycation end products activate NADPH oxidase, generating reactive oxygen species.^[46]^ These reactive oxygen species further activate NF-κB, promoting IL-1β and TNF-α expression and forming a pro-inflammatory oxidative stress loop.^[47]^ The PI3K/Akt pathway exerts dual regulatory effects on cardiovascular homeostasis: it preserves mitochondrial integrity through phosphorylation of pro-apoptotic proteins and also, when hyperactivated, disrupts autophagy and exacerbates oxidative stress via mTOR signaling.^[48–50]^ Aspartame’s potential interference with these tightly regulated pathways may alter cardiomyocyte survival and vascular integrity. Network analysis revealed highly coordinated functional modules involving inflammation, apoptosis, and signal transduction, suggesting that aspartame’s cardiovascular toxicity may arise from concurrent disruptions across multiple key regulatory nodes.

Cardiovascular risk results from the interplay of genetic and environmental factors, with dietary components being major modifiable contributors. Growing concern has emerged regarding the health impacts of artificial sweeteners. Recent prospective studies have linked food emulsifiers to increased CVD risk,^[51]^ and UK Biobank data suggests a correlation between artificial sweetener intake and CVD.^[52]^ These epidemiological findings highlight the scientific necessity of elucidating molecular mechanisms underlying the cardiovascular effects of widely used sweeteners like aspartame.

This study has several limitations. Molecular docking is based on static crystal structures and idealized conditions, which may not fully reflect dynamic in vivo environments. Protein conformational flexibility, membrane interactions, and metabolic conversion are not captured in docking simulations.^[53]^ Furthermore, binding affinity does not directly equate to biological activity or clinical outcomes. Actual biological effects depend on tissue exposure concentration, exposure duration, and cellular sensitivity thresholds, which were not evaluated here.

Additionally, individual metabolic variations can affect aspartame accumulation and sensitivity. Populations with high exposure (frequent consumers of sugar-free products) may face increased risk,^[54]^ while individuals with preexisting CVD may exhibit heightened sensitivity.^[55]^ Functional polymorphisms in key genes such as IL1B, CASP3, and SRC may also lead to differential susceptibility.^[56]^ From a public health perspective, our findings support the implementation of individualized and risk-stratified aspartame usage guidelines. Rather than suggesting a universal ban, these results advocate for precision regulation, particularly among CVD-vulnerable populations. We hope this study stimulates further research to enhance public health protections.

## 5. Conclusion

This study systematically investigated the molecular interactions between aspartame and the cardiovascular system using network toxicology and molecular docking approaches. Key findings include: construction of a cardiovascular-relevant molecular interaction network; identification of IL1B, CASP3, and SRC as hub genes, providing insight into potential molecular targets underlying cardiovascular effects; and prediction of coordinated network effects involving AGE-RAGE, NF-κB, and PI3K/Akt signaling pathways. These results offer a theoretical foundation and experimental direction for future studies on aspartame’s cardiovascular safety. However, further in vitro and in vivo validation is necessary to determine its clinical relevance.

## Author contributions

**Conceptualization:** Taoyu Yang.

**Data curation:** Taoyu Yang.

**Project administration:** Jingjing Wang.

**Supervision:** Lili Zhang, Haowei Li.

**Visualization:** Jiangting Luo.

**Writing – original draft:** Taoyu Yang, Jiangting Luo.

**Writing – review & editing:** Lili Zhang, Haowei Li.
